# Determination of the Electron-Antineutrino Angular Correlation Coefficient a_0_ in Unpolarized Neutron β-Decay

**DOI:** 10.6028/jres.110.059

**Published:** 2005-08-01

**Authors:** J. Byrne

**Affiliations:** Department of Physics and Astronomy, University of Sussex, Brighton BN1 9QH, UK

**Keywords:** antineutrino, cold neutron, correlation coefficient, neutron decay

## Abstract

The coefficient *a*_0_ has been derived from a measurement of the integral spectrum of recoil protons stored in a quasi-Penning trap with inhomogeneous magnetic field and adiabatic focusing onto an electro-static mirror of potential variable in 10 V steps between 0 V and 850 V. Correction for incomplete transfer of energy from transverse to longitudinal degrees of freedom, and the violation of the adiabatic conditions on reflection at the mirror, is carried out by alternately measuring the spectrum at trapping times of 1 ms and 2 ms. The results *a*_0_ = −0.1054 ± 0.0055 and |*λ* | = 1.271 ± 0.018 are comparable in precision with existing measurements of *a*_0_.

## 1. Introduction

The proton and neutron are the lightest members of the lowest flavour SU(3) baryon octet, and neutron β−decay

$$n\Rightarrow {p+e}^{-}+{\bar{\nu }}_{e}$$ (1)

is a strangeness conserving (*Δ S* = 0) semi-leptonic decay, whose rate is governed by the weak vector and axial vector coupling constants *G*_V_ and G_A_. The anomalously long lifetime of the neutron [1], *τ*_n_ = (885.7 ± 0.8) s, is purely a consequence of the extremely low energy release which is less than 0.1 % of the nucleon mass. The decay is interpreted according to the universal V-A theory of weak interactions [2] with a conserved vector current derived from an approximate global symmetry of the QCD Lagrangian [3]. Thus *G*_V_ is expressible in terms of the Fermi coupling constant according to the relation [4 ]

$$G_{v}\left(\Delta S=0\right)=G_{F}\left(1+\Delta_{\beta }-\Delta_{\mu }\right)V_{\text{ud}},$$ (2)

where *Δ*_β_ and *Δ*_µ_ are inner radiative corrections associated with beta and muon decay respectively, and *V*_ud_ is the leading element of the Cabibbo-Kobayashi-Maskawa mixing matrix [3]. Since the axial current is not conserved, *G*_A_ is renormalised by the strong interactions and possibly by non-Standard Model contributions to the weak interactions [2]. It must therefore be determined by experiment. It follows that neutron decay can be characterised by *G*_V_ together with the ratio [1]

$$\lambda =G_{A}/G_{V}=-1.2695\pm 0.0029.$$ (3)

Although *G*_A_ is itself of great theoretical interest, it is the determination of *G*_V_ which attracts the greatest attention because it offers the only method for determining *V*_ud_ and verifying the unitarity of the CKM matrix. *G*_V_ may be determined from the measured ft-values of the pure Fermi 0^+^ – 0^+^ superallowed *β*-transitions within isospin triplets provided the relevant nuclear physics corrections may be applied with confidence [5]. An alternative approach to the determination of *G*_V_ which is independent of nuclear structure effects is offered by neutron decay alone for which the factor *G*_V_^2^[1 + 3*λ*^2^] may be derived from the neutron lifetime as before, and the value of *λ* from observations on the parity-violating neutron-spin electron-momentum correlation coefficient A in polarized neutron decay [6].

## 2. The Electron-Antineutrino Angular Correlation Coefficient *a*

There is, however, another route to the determination of |*λ* | which has not been fully explored, namely the measurement of the electron-antineutrino angular correlation coefficient *a*. This is given in lowest order by the expression [6]:

$$a_{0}=\left[\left(1-\lambda^{2}\right)/\left(1+3\lambda^{2}\right)\right].$$ (4)

Since this is a parity conserving correlation which does not contain interference terms proportional to *λ*, its observation does not require that the neutrons be polarized. It possesses the further advantage, which it shares with the electron asymmetry coefficient A, that it is proportional to the anomaly (| *λ* |−1) rather than to |*λ*| itself.

To date the most successful method for determining *a*_0_ relies on a measurement [7] of the proton kinetic energy spectrum *g*(*E*) which has the form [8]

$$g\left(E\right)=g_{1}\left(E\right)+a_{0}\;g_{2}\left(E\right),0\leq E\leq E_{m}=0.75\;\text{KeV},$$ (5)

where the function *g*_1_(*E*) ≥ 0 reaches a maximum near the middle of the spectrum at about the same point where the function *g*_2_(*E*) changes sign from negative to positive. The behaviour of *g*_2_(*E*) is a reflection of the fact that, for a Fermi (Gamow-Teller) transition, the momenta **p**_e_ and **p**_ν_ are predominantly parallel (anti-parallel). It should be remarked that *a*_0_ as it appears in Eq. (5) is correctly given by Eq. (4).

## 3. Measurement of the Integral Proton Spectrum using an Ion Trap

The experimental technique is based on a modification of the apparatus used to measure the neutron lifetime where protons from neutron decay, of energy ≤0.75 keV, are stored in a quasi-Penning trap aligned along the neutron beam [9,10]. This is formed by the superposition of an axially symmetric ≈5 T magnetic field on a coaxial system of electrodes with ≈1 kV electrostatic barriers at the trap ends. The end electrode facing the detector is designated as the “gate” while the far end electrode is designated as the “mirror.” In the measurement of *a*_0_ the neutron beam was collimated by a 16 mm diameter aperture fixed at the entrance to the cryostat in combination with a 20 mm aperture located 5 m upstream. The possible means by which this apparatus could be employed to determine a value for *a*_0_ have been analysed in detail [11].

**Fig. 1:**
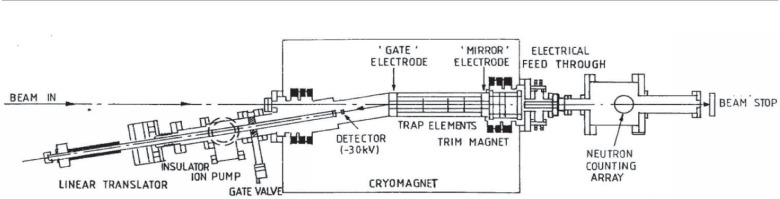
Cryomagnetic ion trap for storing protons from neutron decay. The trim magnet at the beam exit was added to the original apparatus [9] to produce a uniform weak field in this region.

**Fig. 2:**
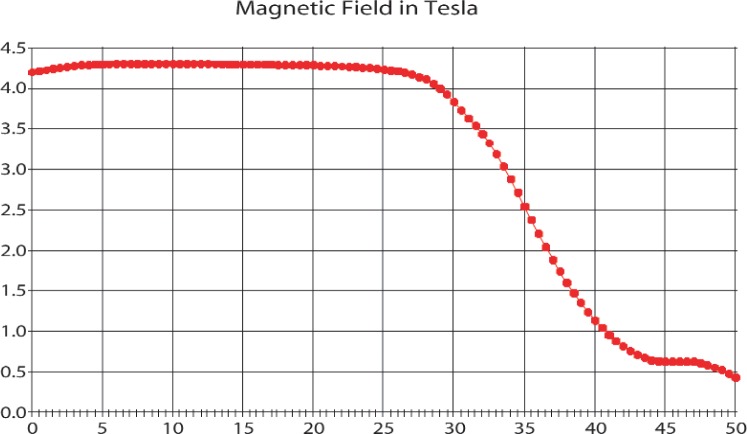
The magnetic field distribution in the trap as a function of axial distance (*z*) in centimeters. The beryllium mirror electrode is fixed at *z* = 46.5 cm. The “long trap” samples the region from the mirror to about *z* = 5 cm, and the “short trap” from the mirror to about *z* = 23.5 cm.

In the simplest method the potential on the gate electrode is kept constant at about 0.85 kV while the mirror electrode may be set at different potentials *V*_0_ in order to measure the number *N*_1_(*V*_0_) of protons trapped behind a barrier of variable height *V*_0_. This is what is meant by the “one-dimensional integrated spectrum,” since protons are trapped if the energy *in their longitudinal degree of freedom* is less than e*V*_0_. However Monte Carlo simulations based on the known spatial variation of the magnetic and electric fields [11] and the theoretical proton spectrum [8] indicated that this spectrum was rather insensitive to the value of *a*_0_. It was therefore found advantageous to exploit the action of a nonuniform magnetic field by establishing the mirror electrode in a region where the magnetic field has fallen to a very *low* value. The modified apparatus and magnetic field map are shown in Figs. 1 and 2.

In this variant, which is the inverse of the normal magnetic mirror effect whereby energy is transferred from the longitudinal to the transverse mode when a charged particle is transported into a region of *high* magnetic field, nearly all the transverse energy is transferred into the longitudinal mode, an effect described as adiabatic focusing or collimation. Thus the measured integrated spectrum coincides very closely with the full three-dimensional spectrum. It is, however, essential that the magnetic field be uniform at the position of the mirror electrode, and to this end a permanent magnet in the form of an annulus has been built into the mirror electrode. Under these conditions, and assuming exact adiabatic invariance, approximately 90 % of the total proton energy should appear in the longitudinal degree of freedom [11]. In order to avoid the necessity for computing the detection efficiency for protons created in the region of inhomogeneous magnetic field, the experiment is carried out with two different trap lengths; a “long” trap and a “short” trap, with the difference in counts giving the number *N*_3_(*V*_0_) of protons created in the region of high uniform magnetic field and trapped in the region of low uniform magnetic field [10]. At the end of each trapping cycle the mirror potential is lowered to zero to permit any protons or electrons which remained trapped to escape on to the mirror.

According to the field plot shown in Fig. 2, the spatial variation of the magnetic field in the middle range where *B*≈2 T is such that the field changes by about 7 % in one period of cyclotron oscillation for a proton of average energy moving with a velocity ≈250 km s^−1^. This change might reasonably be described as adiabatic. However at *B*≈1 T the field change per cycle increases to about 30 % per period and the assumption of exact adiabaticity fails. Thus transverse and longi-tudinal motions are coupled in this region and an oscillating proton visits every state open to it by conservation of energy and angular momentum. The result is that “unbound” protons with energy above the barrier set by the mirror, but with initial longitudinal energy below the barrier, will ultimately escape. However, the time scale must be determined from the experimental data.

## 4. Experimental Results

The first experiments were performed during two cycles of beam-time on the high-flux cold neutron beam PF1 at the Institut Laue-Langevin in Grenoble, France in 1997. The maximum count rate for trapped protons was about 4 s^−1^ with a background/signal ratio less than 1 %. The integral spectra observed in both long and short 10 ms traps were recorded in 50 V steps of *V*_0_ up to a maximum of 900 V with the same statistical error at each point corresponding to about 1 % at the highest counting rate. Approximately 10^6^ events were recorded in total [12].

**Fig.3:**
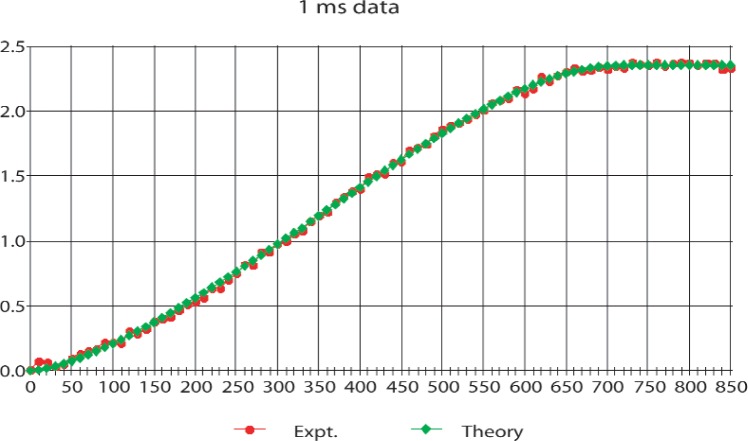
Comparison of experimental data with theory for summed 1 ms runs. The vertical axis shows the integrated counts in arbitrary units and the horizontal axis shows the mirror potential in volts.

**Fig. 4:**
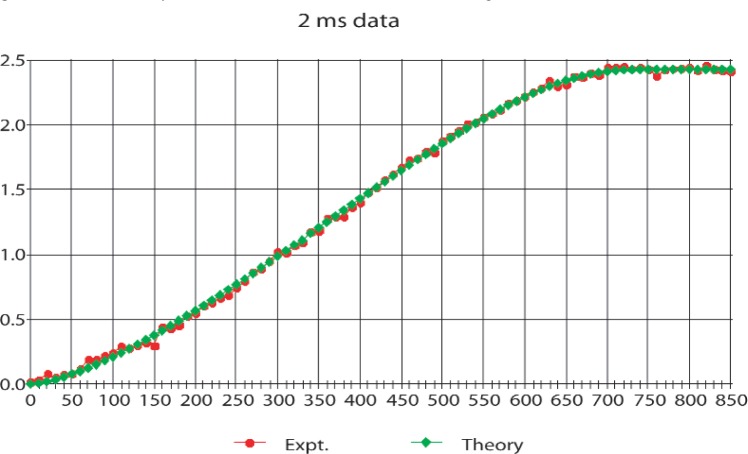
Comparison of experimental data with theory for summed 2 ms runs. The vertical axis shows the integrated counts in arbitrary units and the horizontal axis shows the mirror potential in volts.

The final experiments were performed during two cycles in 1998 and the results including many of the technical details have been published [13]. For this work the counting rates were raised from about 4 s^−1^ to about 20 s^−1^. Thus, to keep the deadtime correction [13] associated with the simultaneous release of two or more protons from the trap at an appropriate level, the trapping time was reduced from 10 ms to 1 ms or 2 ms with a corresponding increase in background which was determined separately. In these runs the mirror setting was altered in 10 V steps with a precision of ± 10 mV, from 0 V up to 850 V with the gate set a fixed value of 850 V. At the end of each data-taking cycle the gate potential was lowered to zero to allow a background count. The results are shown in Figs. 3 and 4.

The results of an analysis based on the *assumption* that all particles which are *not* energy bound escape over a time scale which is negligible compared with the trapping time are displayed in Table 1.

Since the difference between the two mean values of *a*_0_ is non-zero at a level of significance < 0.1 %, the hypothesis of an infinitesimal lifetime for unbound particles in the trap must be rejected. Indeed, since unbound particles are released at a constant rate into the trap, whereas the loss rate is proportional to the number present at any one time, it follows that the number of unbound particles which are trapped must eventually reach an equilibrium value. The difference in the measured values of *a*_0_ may then be used to determine this lifetime, under the weaker hypothesis that the number of unbound trapped particles reaches equilibrium in a time < 1 ms. The results of this re-analysis are shown in Table 2.

The mean value of *a*_0_ derived from all the measurements listed is

$$a_{0}=-0.1054\pm 0.0055$$ (6)

where the error quoted is the standard error on the mean for 10 degrees of freedom. The final value for |*λ* |is obtained by application of Eq. (4) with the result

|λ|=1.271±0.018. (7)

## Figures and Tables

**Table 1:** Results for six runs each at 1 ms and 2 ms trapping times. It is tentatively assumed that “unbound” protons escape in times ≪ 1 ms

Run No.	*a* (1 ms)	std. dev.	*χ*^2^/dof	*a* (2 ms)	std. dev.	*χ*^2^/dof
34	−0.241	0.031	1.58	−0.178	0.021	1.05
35	−0.266	0.024	1.78	−0.189	0.02	1.18
36	−0.212	0.026	1.16	−0.186	0.023	1.23
37	−0.234	0.03	1.65	−0.15	0.024	1.6
38	−0.259	0.028	1.38	−0.172	0.027	1.66
40	−0.262	0.03	1.31	−0.196	0.025	1.44
	<*a* (1 ms)>			<*a* (2 ms> =		
	−0.246	0.009	0.43	−0.179	0.007	0.49

**Table 2:** Values of *a*_0_ and standard errors for six runs each at 1 ms and 2 ms trapping times and a best fit value for the mean lifetime of unbound particles in the trap of (0.303 ± 0.019) ms

Run No.	*a* (1 ms)	std. dev.	*χ*^2^/dof		*a* (2 ms)	std. dev.	*χ*^2^/dof
34	−0.1016	0.027	1.31		−0.1052	0.021	1.02
35	−0.123	0.021	2.02		−0.1158	0.019	1.04
36	−0.0706	0.024	1.28		−0.1117	0.0222	1.09
37	−0.0919	0.029	1.82		−0.0768	0.022	1.45
38	−0.1206	0.028	1.26		−0.0991	0.025	1.62
40	−0.1252	0.031	1.11		−0.1237	0.027	1.4
	<*a* (1 ms)>				<*a* (2 ms)>		
	−0.10548	0.0054	0.75		−0.10538	0.00668	0.65
		<*a*> =	−0.1054	+/−0.0055	*χ*^2^/dof = 0.63		
